# Supplementary material for: Protection of Phage Applications in Crop Production: A Patent Landscape
Source: Viruses. 2019 Mar 19;11(3):277. doi: 10.3390/v11030277 (PMC6466637; doi:10.3390/v11030277)
Supplement: Supplementary file 1 [file viruses-11-00277-s001.zip › viruses-458590 final supplementary/viruses-458590 revise supplementary.pdf]

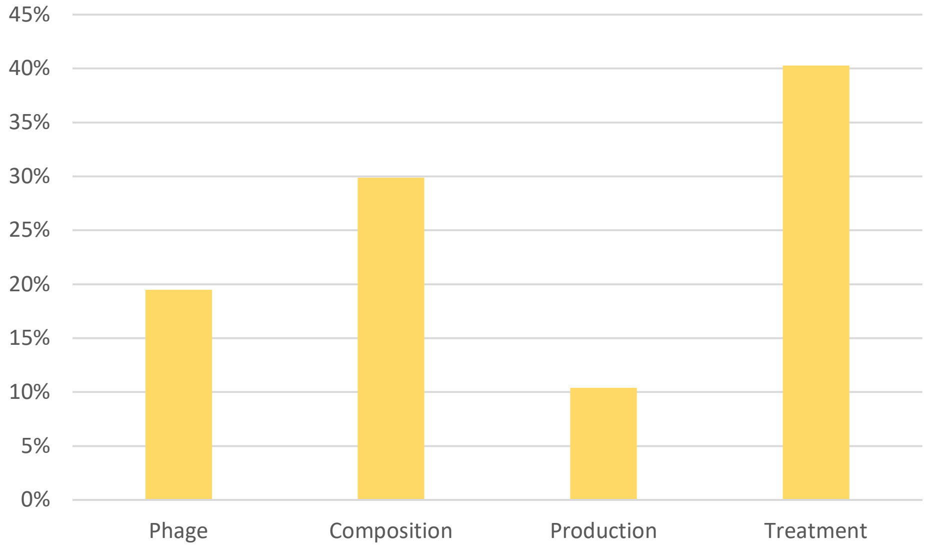

Supplementary Figure S1 Percentages of independent claims per category. The independent claims, 77 in total, from the 21 granted patents have been categorized among four different categories: (1) Phage – here the phage was described as the active ingredient or the isolation of a phage was described, (2) Cocktail – this category contains claims that protect the combination of phages or phages as part of a composition, (3) Production – ways of how the phage is produced and (4) Treatment – claims that protect the use of phages to fight a specific bacterial infection or methods and application strategies for using phage.
